# Gaultheria in Rheumatism

**Published:** 1883-10

**Authors:** 


### Gaultheria in Rheumatism.

At a meeting of the New York Medical and Surgical Society, Dr. Flint said that one of the house physicians at Bellevue Hospital had furnished him with the following statistics with regard to the use of gaultheria in that institution in cases of articular rheumatism (*New York Medical Journal*). Of thirteen cases thus treated of which the histories were given, one patient contracted pneumonia after the cure of the rheumatism, and died in the hospital; a second one remained in the hospital, at the expressed wish of the commissioners some time after cure of the remaining eleven cases; the longest duration of the disease in any one case was fifteen days, and the shortest two days. The average length of time the eleven patients remained in the hospital was a fraction less than five days. These figures would seem to point to rather better results from the drug than those which were ordinarily obtained from salicylic acid. The oil of wintergreen was the preparation used, and it was administered several times a day in ten drop doses in flax-seed tea, which made it less disagreeable to the taste and to the stomach. In some of the cases the alkaline treatment was employed at the same time with the gaultheria.—*Weekly Medical Review.*

